# Thermal Nanoimprint Lithography—A Review of the Process, Mold Fabrication, and Material

**DOI:** 10.3390/nano13142031

**Published:** 2023-07-08

**Authors:** Noriyuki Unno, Tapio Mäkelä

**Affiliations:** 1 Department of Applied Electronics, Tokyo University of Science, 6-3-1 Niijuku, Katsushika-ku, Tokyo 125-8585, Japan; 2 VTT Printed and Hybrid Functionalities, Tietotie 3, P.O. Box 1000, FI-02044 VTT Espoo, Finland; tapio.makela@vtt.fi

**Keywords:** thermal nanoimprint, seamless roll mold, replica mold, roll-to-roll, compostable films

## Abstract

Micro- and nanopatterns perform unique functions and have attracted attention in various industrial fields, such as electronic devices, microfluidics, biotechnology, optics, sensors, and smart and anti-adhesion surfaces. To put fine-patterned products to practical use, low-cost patterning technology is necessary. Nanoimprint lithography (NIL) is a promising technique for high-throughput nanopattern fabrication. In particular, thermal nanoimprint lithography (T-NIL) has the advantage of employing flexible materials and eliminating chemicals and solvents. Moreover, T-NIL is particularly suitable for compostable and recyclable materials, especially when applying biobased materials for use in optics and electronics. These attributes make T-NIL an eco-friendly process. However, the processing time of normal T-NIL is longer than that of ultraviolet (UV) NIL using a UV-curable resin because the T-NIL process requires heating and cooling time. Therefore, many studies focus on improving the throughput of T-NIL. Specifically, a T-NIL process based on a roll-to-roll web system shows promise for next-generation nanopatterning techniques because it enables large-area applications with the capability to process webs several meters in width. In this review, the T-NIL process, roll mold fabrication techniques, and various materials are introduced. Moreover, metal pattern transfer techniques using a combination of nanotransfer printing, T-NIL, and a reverse offset are introduced.

## 1. Introduction

### 1.1. Demand for Micro/Nanopatterning

Micro- and nanopatterned surfaces are highly useful as functional surfaces, as well as in the fabrication of electronic devices. For example, to realize artificial intelligence systems, improving the performance of the central and graphic processing units is essential. Based on Moore’s law for semiconductor devices, the limit to the miniaturization of the transistor size will soon be reached. To achieve advancements beyond Moore’s law, monolithic three-dimensional (3D) integrated circuits are widely investigated [1]. Developing nanopatterning techniques for large areas is essential to reduce the cost of nanopatterning on a wafer scale [1].

Micro-total analysis systems (µ-TAS) [2] and biotechnology-like cell cultures [3] also require micro- and nanopatterned surfaces. Nanopatterned surfaces are used to fabricate a hydrophilic channel that easily transports liquids. In addition, a disposable package is required for these applications. Hence, a low-cost fabrication technique is strongly desired.

Optical applications widely utilize micro- and nanopatterns. For example, micro-lens arrays are essential for improving the performance of image sensors [4]. Anti-reflection films with nanostructures reduce the reflection of light in display devices [5] and improve the power generation efficiency of solar cells [6].

Moreover, many sensor applications utilize micro- and nanopatterns, and fine metal patterning is indispensable for these applications. Touch sensor panels, which can transmit light emitted from a display device, consist of a micro-scale metal wire on a transparent substrate [7]. To obtain a highly sensitive chemical sensor, surface-enhanced Raman scattering (SERS) using a nanodot pattern made of metal is a promising technology [8].

Printable electronics have important applications in electricity generation, particularly in organic solar cells [9] and thermoelectric devices [10] that require large-area micropatterns. In printable thermoelectric devices, reducing electrical contact resistance [11] and precise aligning techniques for p-n junctions are crucial, enabling scalable and cost-effective production.

Smart surfaces and anti-adhesion surfaces in facilities help reduce the cost of maintenance such as cleaning, defrosting, and disinfection. For example, bacterial anti-adhesion surfaces are needed to prevent biofilm formation [12]. A surface inspired by the nanostructures on insect wing surfaces is used for anti-bacterial and self-cleaning surfaces [13]. Frost formation and its impact on the icephobic properties of superhydrophobic surfaces have also been studied [14].

As noted above, micro- and nanopatterns are necessary for next-generation devices in various fields. A high throughput patterning technique with design flexibility, including 3D patterns, is highly desired. Fine patterns can be obtained by cutting [15], laser beam machining [16], electron beam lithography (EBL) [17], and focused ion beam (FIB) milling techniques [18]. Photolithography using extreme ultraviolet (UV) light is the most powerful technique for nanofabrication to date [19]. The interference lithography technique facilitates regular nanopatterns over a large area with no reticle [20]. Self-assembled patterning methods are expected to be low-cost means of nanofabrication because expensive machines are not required. Table 1 shows the specifications of various fine patterning techniques. Consequently, future fine patterning techniques are anticipated to offer low cost and high throughput with design flexibility over a large area.

### 1.2. Basis of Nanoimprint Lithography

To meet the demand for next-generation fine patterning techniques, NIL was developed in 1995 by Chou et al. [21]. Here, a nanopatterned mold was prepared by EBL. Using the mold, a poly(methyl methacrylate) layer was imprinted on a silicon substrate. The principle of NIL is similar to the conventional method of hot embossing. However, because pattern sizes as small as 25 nm can be obtained using the NIL process, it has received much attention to date. The concept of NIL combines a high-resolution technique, such as EBL, with a high-throughput pattern transfer process. In 1996, Haisma et al. developed the ultraviolet NIL (UV-NIL) process [22], which uses a photo-curable resin. The high-speed curing time of the UV-NIL process allows for very high throughput. Moreover, the viscosity of UV-NIL resin is comparatively low. Thus, it can be used to fill a very fine mold, with patterns as small as 10 nm [23]. However, UV-NIL resins are typically expensive because they contain a highly designed monomer, photoacid generator, and solvent to reduce viscosity. Moreover, these chemicals must be handled with care, and equipment for volatile organic compound (VOC) treatment is necessary. Importantly, the substrate must tolerate UV exposure. The differences between the conventional patterning and NIL processes are shown in Table 2.

The original NIL process is commonly referred to as thermal nanoimprint (T-NIL) because it uses a thermoplastic resin. In principle, the throughput of T-NIL is lower than that of UV-NIL because T-NIL requires time for heating to reach a low viscosity and cooling. However, T-NIL offers the advantages of lower material cost compared with UV-NIL, and an eco-friendly process that does not require VOC chemicals. Therefore, in this review, recent progress regarding T-NIL in the development of the process (Section 2), mold fabrication including roll molds (Section 3), and materials (Section 4) are discussed to outline possible future directions of T-NIL.

## 2. Various T-NIL Processes

### 2.1. Planar T-NIL

**Figure 1:**
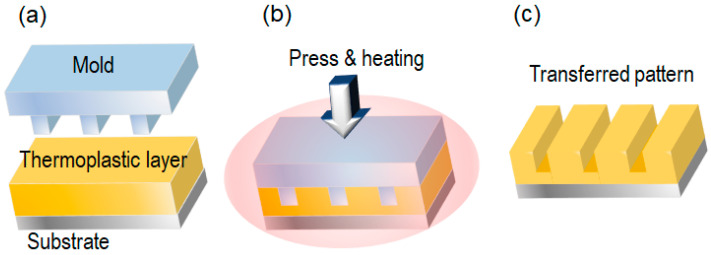
Thermal nanoimprint (T-NIL) process with a planar mold [25]. (**a**) alignment; (**b**) press and heating; (**c**) mold releasing.

Figure 1 depicts the T-NIL process using a planar mold [25]. The mold was fabricated by EBL and made of silicon wafers. First, a thermoplastic layer was formed on a Si substrate by spin-coating. Second, a mold was placed on the substrate. The mold was pressed against the substrate while being heated to its glass transition temperature (*T_g_*), and the thermoplastic layer was deformed according to the mold pattern. Then, the mold and substrate were cooled to less than *T_g_*. Finally, the mold was released from the substrate. Typically, there is a residual layer under the transferred pattern, which must be removed by a plasma etching process to perform the lift-off and an etching process for the substrate. For example, 25 nm diameter and 120 nm period metal dots were fabricated by planar T-NIL and a lift-off process.

**Figure 2:**
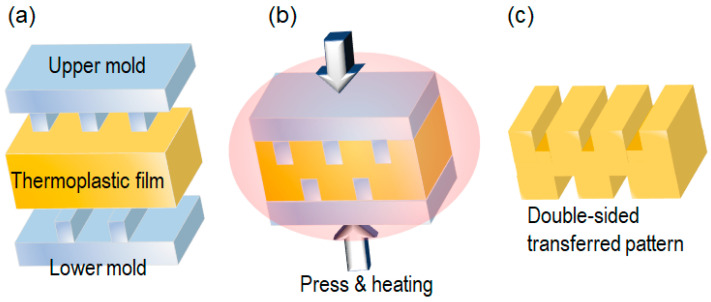
Double-sided T-NIL process using two planar molds. (**a**) alignment; (**b**) press and heating; (**c**) molds releasing.

In the double-sided process, the substrate is replaced by another mold, as shown in Figure 2. In this process, although the thermoplastic film should provide enough thickness for handling, the transferred patterns are obtained on both sides of the film [26]. Only the surface of the film can be heated to or near its *T_g_*. Heating the entire film above or close to *T_g_* would cause deformation or stretching. The double-sided patterned film is particularly valuable for optical applications. This process can also be applied to thermosetting plastics.

**Figure 3:**
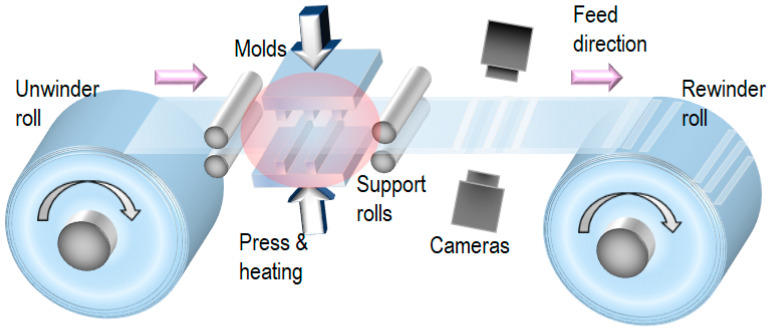
T-NIL process using planar molds with a roll-to-roll web system.

To enhance the throughput of T-NIL, a roll-to-roll web system [27] can be employed, as depicted in Figure 3. The conventional silicon wafer process requires the use of a very expensive wafer transfer robot because silicon wafers are currently incompatible with a web system. By contrast, thermoplastic films are flexible and compatible with the web system. This process allows for the easy combination of T-NIL with the web system. Although planar molds are readily available, the throughput of this system is limited by the time required for heating and cooling during the T-NIL process. Nevertheless, detailed inspection of transferred patterns using cameras is possible. In addition, web tension-related issues are irrelevant in this process as the web is easier to heat stabilize.

### 2.2. Roller T-NIL

**Figure 4:**
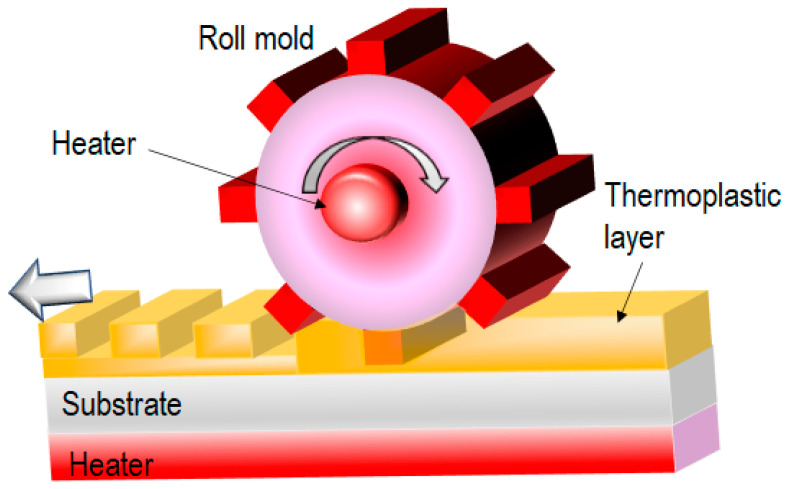
Roller T-NIL process using a roll mold [28].

Obtaining planar NIL molds using a conventional lithography process with EBL or photolithography is easy. However, these molds are typically made of silicon wafers or quartz, which are hard materials. A roller T-NIL process using a roll mold [28] is suitable for overcoming the bottleneck of the heating and cooling times in T-NIL processes (Figure 4). Because the roll mold is difficult to fabricate, a replica mold made by nickel electroplating is widely used for roller T-NIL. Here, nickel foil, used for its flexibility, is attached to a roll substrate, and the nickel replica mold has sufficient temperature durability for the T-NIL process. Therefore, the mold pattern can be transferred to the thermoplastic layer coated on a planar substrate, eliminating the need for prolonged heating and cooling. The transferred thermoplastic layer can be fabricated by spin-coating, allowing for sub-µm layer thickness, which can be easily removed by etching after the lift-off process. In this case, tension control for web feeding is not necessary. A softer backing roll helps to increase the contact time between the roll mold and the thermoplastic layer. However, precise temperature, speed, and pressure control are necessary because cooling is achieved naturally and without cooling equipment. In some instances, the pattern can be achieved through the plastic deformation of the thermoplastic layer.

### 2.3. Roll-to-Roll T-NIL

**Figure 5:**
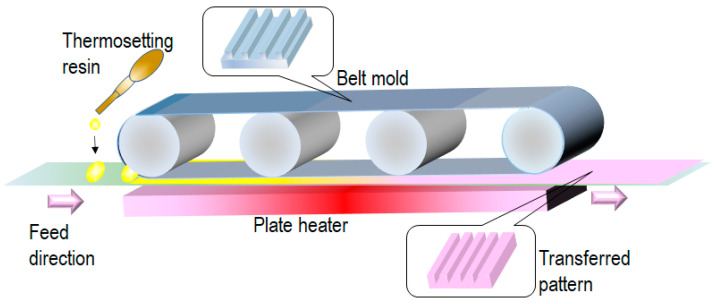
Belt-type T-NIL process using a thermosetting resin [29].

To further improve the throughput and transferred pattern quality, belt-type T-NIL processes have been proposed [29,30]. These processes require a belt-type flexible mold. A replica mold is widely used for this purpose. Figure 5 shows the schematics of the belt-type T-NIL process using a thermosetting resin such as polydimethylsiloxane (PDMS). A plate heater is used to cure the thermosetting resin. The heater does not require contact with the base film because the tension of the base film facilitates the contact force between the belt mold and the base film. For example, when PDMS is used for thermosetting resin, the curing time is 2–3 min at 130 °C. In this case, a flexible polyurethane acrylate mold is used.

**Figure 6:**
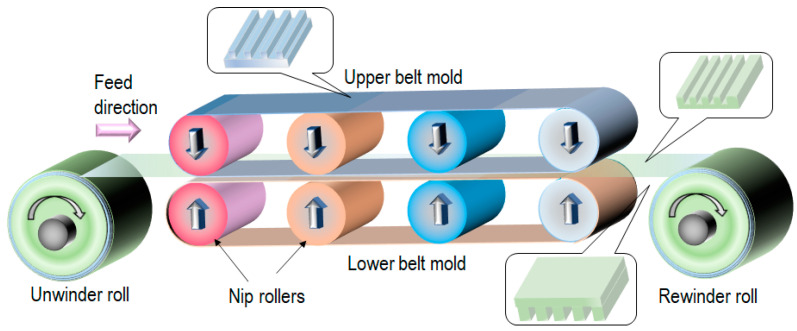
Belt-type T-NIL process using a thermoplastic film [30].

Figure 6 shows the schematics of the belt-type T-NIL process using a thermoplastic film. In this process, the cooling process is crucial to improve the throughput. If the imprinted pattern is released from the mold at a temperature above *T_g_*, the pattern collapses immediately. Thus, cooling rollers or additional cooling equipment are essential for high throughput. In Ogino, et al., dots with a diameter of 200 nm and a height of 240 nm were formed on a 15-meter-long polystyrene sheet within 27 min [30].

**Figure 7:**
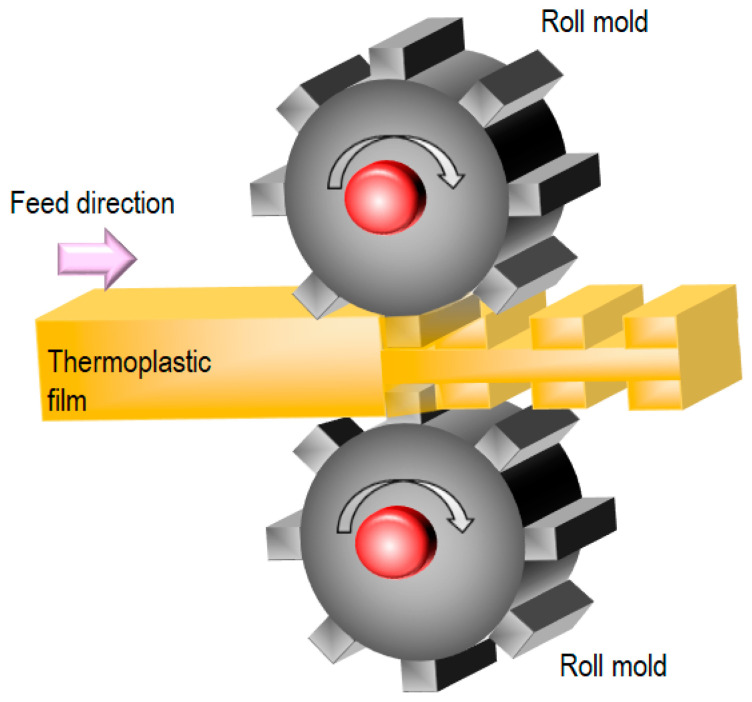
Roll-to-roll T-NIL process using a thermoplastic film [31].

Figure 7 illustrates the roll-to-roll T-NIL process using a thermoplastic film [31]. As in other processes using a roll mold, nickel replica molds are attached to rolls equipped with internal heaters. In this process, a thermoplastic film is directly fed into the gap between the roll molds. Alternatively, a pre-heating system is applied before the film is fed into the gap. Because there is no cooling equipment, the system configuration is very simple, resulting in a low unit cost. The mechanism of pattern transfer is mainly based on plastic deformation. Thus, precise temperature control with appropriate pressure is important for obtaining transferred patterns. The contact length in roll-to-roll NIL can be varied by the use of softer backing rolls, providing a longer contact time. For example, a thermoplastic film measuring 95 mm in thickness and 50 mm in width was structured using this method.

### 2.4. Nanotransfer Printing (nTP)

**Figure 8:**
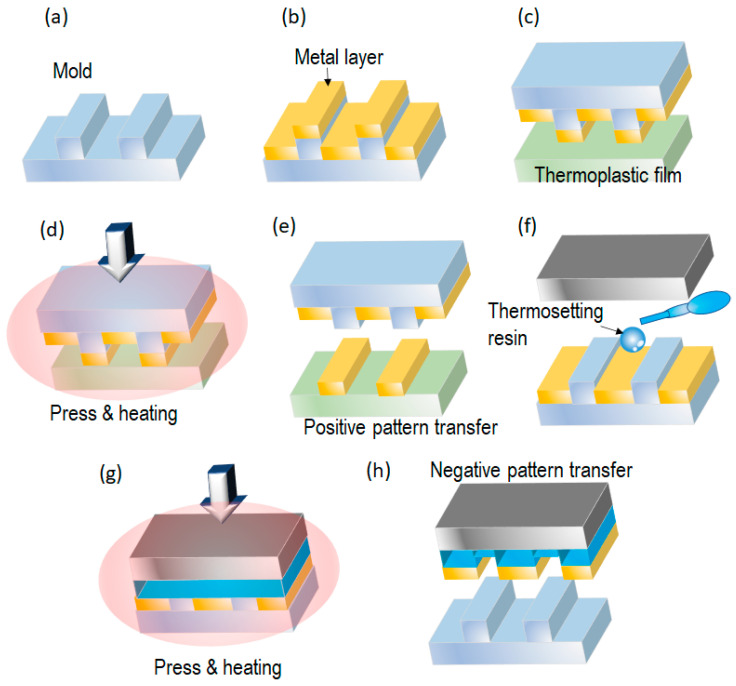
Nanotransfer printing (nTP) process (**a**–**e**) [32] and two-tone nTP (**f**–**h**) [33].

The above T-NIL processes focus on the fabrication of plastic or resin patterns. However, some sensors, such as SERS sensors, require metal nanopatterns on a plastic substrate. Nanotransfer printing (nTP) [32] is a promising technology for fabricating metal patterns on plastic substrates. The original nTP process is shown in Figure 8a–e. First, a mold is coated with a transferred metal. Next, the coated mold and a plastic substrate are pressed against each other under heating. The plastic substrate is not deformed by the pressure, and only the metal layer on top of the mold is in contact with the plastic substrate. Using the difference in surface tension, the top metal layer is transferred to the plastic substrate. When the mold is pressed against the plastic substrate at high pressure, all metal layers are transferred.

Two-tone nTP has been proposed as an alternative nTP method [33] (Figure 8f–h). Here, after the normal nTP process (positive-tone pattern), the metal layer in the bottom of the mold is transferred using a thermosetting resin (negative-tone pattern). After the nTP process, the mold can be utilized repeatedly. The combination of nTP and roll-to-roll T-NIL realizes eco-friendly metal nanopatterning with high throughput because it does not require solvents and chemicals for lift-off and metal etching.

### 2.5. Reverse Offset Technology

**Figure 9:**
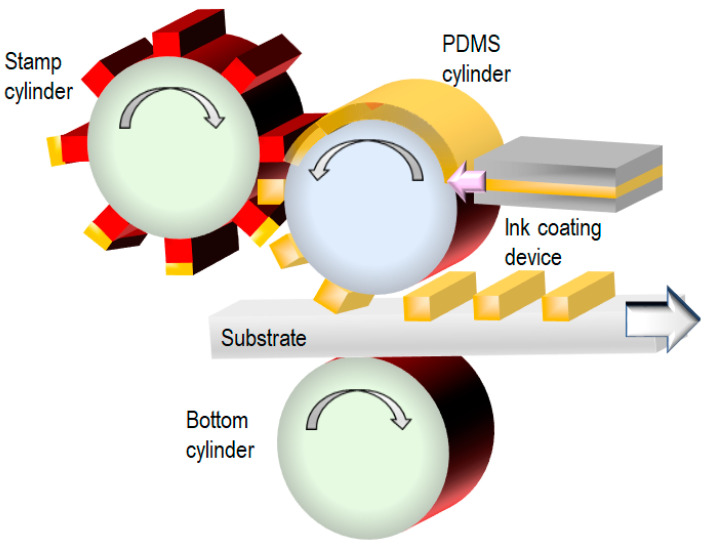
Reverse offset (RO) process for roll-to-roll metal patterning [34].

nTP typically uses a metal layer deposited by physical vapor deposition, which requires a long time to prepare a vacuum condition. As a result, obtaining the metal layer on a large area is difficult. By contrast, reverse offset (RO) technology [34] provides high throughput and high resolution (1 µm) using conductive nanoparticle inks (Figure 9). Furthermore, a roll-to-roll process for RO has been achieved. In this method, control of the surface energy is crucial for the ink pattern transfer. 

## 3. Seamless Roll Mold Fabrication Technique

In principle, the size of the transferred patterns obtained by T-NIL processes depends on the features of the mold. Therefore, the fabrication technique for NIL molds is crucial. Planar NIL molds are typically obtained by photolithography or EBL. However, increasing the throughput of planar T-NIL processes is challenging. Consequently, roll mold fabrication techniques are essential for next-generation T-NIL processes. In particular, seamless roll molds increase the product yield by eliminating wasted space on the transferred substrate caused by seams. In this section, various fabrication techniques for the seamless roll mold are described.

### 3.1. Advanced Machining Technology

Machining processes have a long history and are the most common technique for micro- and nanopatterning. Single-point diamond turning is a promising method for obtaining a desired pattern on a roll. The diameter of the diamond tip determines the obtained pattern size. Sun et al. applied a focused ion beam to sharpen diamond tips [35]. The mouth width of the machined pattern with the sharpened tip was 447 nm, while its depth was 607 nm. An indenting method using a diamond die was reported by Cates et al. [36]. However, using nanoscale tips or dies for long periods is challenging. Electrical discharge machining (EDM) is an efficient method for micro- and nanopatterning in which the wear of tools is not a concern. For example, wire EDM was performed to fabricate roll molds [37]. However, because the wire diameter was 250 µm, the main pattern width was not nanoscale. On the other hand, EDM can also produce a surface with multi-scale roughness that is applicable as a superhydrophobic surface. Laser beam machining [38], which needs no fine tools or wire, is also useful for obtaining micro-patterns with surface treatment.

### 3.2. Direct Laser Beam Writing

Direct laser beam writing (DLW) techniques have attracted much attention for obtaining a fine roll mold in air [39]. The difference between direct laser machining and DLW is the latter’s utilization of photoresists. Two-photon direct-laser-writing is a powerful method for sub-nm focusing [40]. The two-photon technique enables the fabrication of three-dimensional (3D) patterns in a single process. Furthermore, stimulated emission depletion DLW results in a pattern line width of 65 nm [41]. Although these methods use a point beam to delineate a designed pattern, laser interference lithography [42] can expose light over a larger area. While the design of interference patterns is difficult, this approach helps improve the writing speed of regular patterns, such as line and space patterns.

### 3.3. Direct Electron Beam Writing

The minimum pattern width of normal DLW is determined by the diffraction limit. Electron beam (EB) lithography is a promising technique that can potentially obtain a sub-10 nm pattern. Taniguchi et al. presented a rotating stage to write a designed pattern onto a roll substrate [43]. Similar to the conventional EBL process with a planar substrate, metal lift-off is possible with a roll substrate [44]. However, EBL suffers from a slow writing speed and requires vacuum conditions. Therefore, fabricating a large roll mold by direct EB writing in a vacuum chamber is challenging. To overcome this, an enlargement process from a small roll to a large roll mold was investigated [45]. 

### 3.4. Self-Assembled Patterns

Self-assembled patterning using anodic aluminum oxide (AAO) [46] and block copolymers [47] is a low-cost nanofabrication technique because it does not require expensive machines. In particular, nanopatterns obtained by AAO facilitate anti-reflection patterns of visible light [48]. Furthermore, the AAO process is adaptable to large-roll molds. Therefore, anti-reflection films can be obtained by roll-to-roll nanoimprints with AAO roll molds. Another method using a self-assembled process is to utilize etching. For example, nanopore-type black silicon is fabricated by one-step silver-assisted chemical etching [49]. Oxygen-plasma etching processes with a glassy carbon substrate [50] present a simpler method because this process does not require rare metals and chemicals. Additionally, a 1.5 m long glassy carbon roll mold with a moth-eye structure was achieved [51]. This large-scale roll mold is useful for continuously producing anti-reflection films via the roll-to-roll process.

## 4. Key Technology for T-NIL

### 4.1. Viscoelastic Behavior (N-Curve)

**Figure 10:**
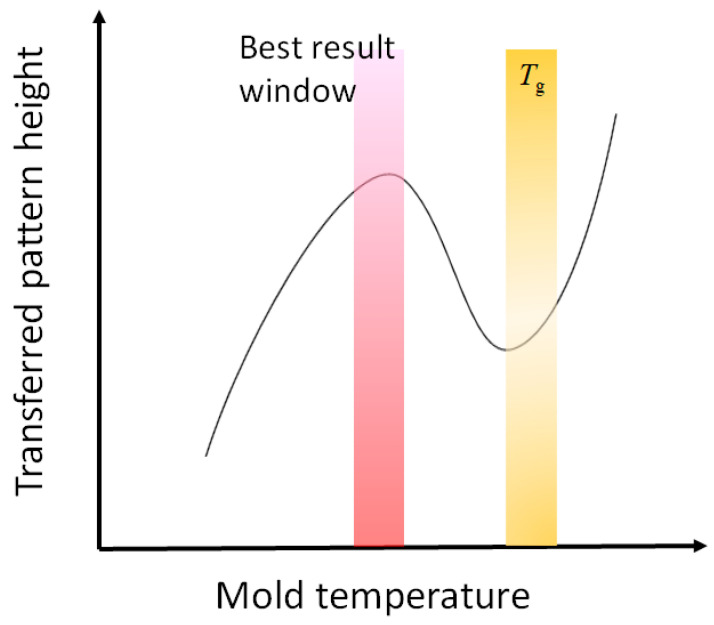
N-curve of the transferred pattern via the roll-to-roll T-NIL process [53].

Roll-to-roll T-NIL with roll molds requires the surface temperature of the mold to be less than *T_g_*. This is because higher temperatures cause the web film to stretch after the nip rollers, resulting in the breakage of the thermoplastic film. Therefore, the transferred pattern is obtained by plastic deformation. Notably, self-relaxation of the transferred pattern is observed when the temperature is significantly lower than *T_g_* [52]. Moreover, at temperatures close to *T_g_*, the viscoelastic property of the thermoplastic film is dominant, causing failure of the mold filling [53]. This phenomenon is commonly referred to as the N-curve of the transferred pattern (Figure 10). Ultimately, to achieve the roll-to-roll T-NIL process using roll molds, precise temperature control of the mold (within the target temperature ±5–10 °C) is essential. 

### 4.2. High-Speed Heating and Cooling Method

The heating and cooling times limit the throughput in the T-NIL process. In particular, the heating method is critical to improving the throughput. Hence, a fast T-NIL employing an integrated heater [54], with a total process time of 10 s, was proposed. Laser-heated rolling T-NIL with a roll-to-roll system was demonstrated [55]. Additionally, induction heating was used to heat a nickel mold containing nanohole arrays [56]. The processing time for a 4-inch diameter scale was less than 5 min, and these heating processes aim to reduce thermal capacity. To overcome the thermal cycle time in T-NIL, assisted heating for ultrasonic nanoimprints [57] were also proposed. 

### 4.3. Replica Mold for High-Temperature Durability

As described above, nickel electroplating is widely used to obtain replica molds for roll-to-roll T-NIL. However, electroplating requires a significant amount of time, resulting in a high cost. In addition, treatment of the waste solutions of nickel plating is troublesome. Consequently, replica molds with high-temperature durability and release properties suitable for the T-NIL process are required. PDMS is typically used for replica molds in T-NIL. However, it is a thermosetting resin and thus requires a long time to cure. Hence, organic-inorganic hybrid resins [58] using a release agent were proposed. Because these resins can be cured by UV light, their duplication time is typically shorter than that of PDMS. However, it requires a release agent to prevent the sticking of the transferred pattern. A UV-curable resin used for replica molds with no release agent [59,60] was also studied. Moreover, the roll-to-roll T-NIL process was demonstrated with the replica mold [61].

### 4.4. Film Preparation and Web System in Roll-to-Roll T-NIL

In roll-to-roll T-NIL processing, film quality plays a crucial role. Common thermoplastic polymers are used, and various parameters such as softening temperature, melting temperature, glass transition temperature, the viscosity of the melted polymer, stringing coefficient, surface energy, bendability, and film thickness affect the replication quality. 

The thermoplastic properties of the film determine the maximum temperature that can be used; however, increasing the film thickness makes it possible to achieve temperatures close to *T_g_* if the processing time is kept sufficiently short. 

In some cases, the surface energy of the thermoplastic material may be insufficient because of the “flow in capillarity” forces caused by the film itself. From a successful process perspective, the film must be flexible enough to be guided from one roll to another. High thermal expansion of the film may potentially impact the desired size of the pattern; however, this can be accounted for in the design of the mold.

One interesting category of future materials is recyclable or even compostable films. For example, novel films made of nanocellulose have unique features such as high transparency, high-temperature tolerance, and, surprisingly, nanoimprintability. These wood fiber-based materials consist of selected nanometer-scale fibers whose film properties can be tuned to improve foldability or printability. In the case of films made of cellulose nanofiber (CNF), the printed structure remains intact even when the moisture content of the film increases, unlike conventional paper. CNF films can be thermally nanoimprinted [62] or imprinted with the assistance of moisture [63]. The increase in moisture content softens the film’s surface, making it easier to form patterns during the imprinting process.

As previously mentioned, web tension and aligning methods are essential in high-speed roll-to-roll T-NIL. In the printing industry, many feedback methods are used. These methods consider web tension and optimize it in situ. Tension is critical for avoiding web stretching or misalignment and ensuring accurate and reliable patterning.

## 5. Materials for the T-NIL Process

The most significant advantage of T-NIL is the flexibility of the transferred material and its environmental properties in practical use: temperature durability, transparency, chemical durability, and mechanical strength (Table 3). In this section, materials for T-NIL and the process conditions are surveyed. Two types of materials were mainly used: thermosetting resin and thermoplastic resin. Super engineering plastics attract considerable attention as well, because of their high temperature and mechanical durability. 

Poly(methyl) methacrylate (PMMA) is commonly used in optical applications because of its high transparency. However, its *T_g_* is relatively low, typically below 100 °C. Polystyrene is known for its radiation resistance, making it suitable for applications such as Petri dishes and food containers. On one hand, polyethylene terephthalate (PET) is transparent and offers excellent gas barrier properties but has limited resistance to acids, alkalis, and chemicals. On the other hand, polyethylene naphthalate shares similar properties with PET but exhibits higher thermal durability and hydrolysis resistance. Polyethylene (PE) is known for its high chemical durability and low density compared with other plastics. PE also has a low brittle temperature (around −40 °C). Polypropylene (PP) also exhibits high chemical durability and has a higher hardness than PE. However, PP is prone to turning white when exposed to sunlight, indicating poor weather resistance. Cyclic olefin copolymer is valued for its transparency and high refractive index (>1.5), making it suitable for optical lenses. Polycarbonate is known for its excellent impact resistance and self-extinguishing properties. Polyether ether ketone is well known for its exceptional thermal durability (>250 °C) and mechanical properties. It is also suitable for use in water and steam conditions. Polyimide (PI) and polyetherimide (PEI) exhibit high thermal resistance (>300 °C for PI and >170 °C for PEI). However, PEI is more cost-effective compared with PI. Both PI and PEI are transparent materials, although they tend to have a yellowish color. Fluorinated ethylene propylene (FEP) and ethylene tetrafluoroethylene (ETFE) are thermoplastic resins used in various applications. Compared with polytetrafluoroethylene (PTFE), which has excellent chemical durability and low surface tension, the viscosity of melted FEP and ETFE is low. Therefore, FEP and ETFE are available for molding, while PTFE requires a cutting process. Cellulose acetate (CA) is a transparent and biodegradable material. In addition, the UV light resistance of CA is high. Polysulfone and polyethersulfone are commonly employed in medical applications such as dialysis membranes because of their excellent mechanical properties and thermal durability, allowing for autoclave sterilization. Polyvinyl chloride is known for its good weather resistance but has relatively low impact resistance. Furthermore, eco-friendly transparent materials such as CA and CNF are essential for sustainable development.

## 6. Conclusions and Overview of the Next-Generation T-NIL Process

Various T-NIL processes are introduced, and seamless roll mold fabrication techniques are reviewed. Planar T-NIL is suitable for silicon- or quartz-based processes because of the rigid nature of these materials. By contrast, roller or roll-to-roll type T-NIL is very compatible with fabricating nanopatterns of plastic substrates. In particular, using a T-die extrusion system facilitates continuous nanopatterned production. Because the belt-type roll-to-roll system contains many rolls, it enables easier film temperature control. However, more rolls increase the equipment cost. To date, various fabrication techniques for seamless roll molds have been investigated (as shown in this review). Seamless roll molds are readily available. Flexible replica molds are a suitable choice when the presence of a seam is not an issue in the final products, such as when using a µ-TAS sheet as an individual package. These molds can be easily attached to a roll substrate, offering the advantage of lower mold costs. Consequently, roll-to-roll T-NIL with roll molds is suitable for mass production because of its low equipment cost.

Although the N-curve of the transferred pattern is of concern, precision temperature control of the roll mold can address this issue. To achieve a stable temperature on the curved surface of a roll mold, it is crucial to develop a reliable temperature measurement method. As noted above, flexible nickel or resin replica mold is typically used; however, forming an emissivity coating layer on the replica mold for radiation thermometers is challenging. Therefore, calibration techniques for the measured temperature on a roll mold are required to ensure accurate temperature monitoring.

In the future, a higher throughput roll-to-roll T-NIL process will be needed. Not only local heating methods but also a local active cooling method should be developed. In particular, for non-uniform patterns in large mold areas (e.g., mixed patterns with dense and sparse areas), a new local temperature control method is required to regulate temperature distribution effectively.

In the roll-to-roll T-NIL process, the significant difference in the thermal conductivity between metal roll molds and thermoplastic films helps to generate a temperature gradient in the film, thus preventing deformation. However, polymer replica molds have similar thermal properties to thermoplastic films. Consequently, a new replica mold made of a polymer with a high thermal conductivity should be investigated to reduce mold cost and increase the throughput in roll-to-roll T-NIL. A high-temperature release agent is required for applications involving increased aspect ratios of transferred nanopatterns.

Moreover, to facilitate future sustainable development, such as smart-built environments and wearable systems [84], the T-NIL process for biodegradable plastics must be investigated further.

## Figures and Tables

**Table 1:** Fine patterning techniques.

Method	Pattern Size	Pattern Flexibility	Equipment Cost	Area Size	Aligning Possibility /Accuracy	Multilayering Possibility	Throughput
Mechanical Cutting	Sub-µm	Very high	Medium	Medium	Sub-micron	Difficult	Low
Direct writing (Laser)	Sub-µm	Very high	High	Small	Sub-micron	High	Low
Direct writing (EB and IB)	>nm	Very high	High	Small	Nanoscale	Possible (IB deposition)	Low
Photo lithography	>10 nm	High	Extremely High	Large	Nanoscale	Difficult	High
Self-assembled patterning	>10 nm	Low	Very low	Very large	Difficult	No	High
Future desired technique	>nm	Very high	Very low	Very large	Nanoscale	High	Very high

**Table 2:** Differences between the conventional patterning process and the NIL process.

	Hot Emboss	Injection Molding	T-NIL	UV-NIL
Pattern size	>Sub-µm	>Sub-µm	>nm	>nm
Applicability	Thermosetting/ Thermoplastic resin	Thermosetting/ Thermoplastic resin	Thermosetting/ Thermoplastic resin	UV-curable resin
Material cost	Low	Low	Low	High
Area	Depends on the heated mold size	Depends on the heated mold size	Depends on the heated mold size	Restricted by the size of the UV exposure field
Mold	Typically hard	Typically hard	Hard/tractile/flexible	Hard/soft/flexible
Throughput	Low	High	Low (Planar process)	Very High
Theory Technological base	Based on continuum mechanics		Based on molecular dynamics [24], like an intermolecular force, surface tension.	

**Table 3:** Materials for T-NIL and process conditions.

Material Name	T-NIL Type	Process Temperature (°C)	Pressure (MPa)	Force (N)	Process Time	Ref.
PMMA	Planar	200	10		3 min	[64]
PS	Planar	160	10		3 min	[64]
PS	Belt-type RTR	120–150	1.2		0.57 m/min Press time 1 s	[30]
PEN	Planar	290	2.5		10 min	[65]
PE	Planar	140	2.7		N/A	[66]
PP	Planar	165–225	5.0		5 min for melting, 30 min for press	[67]
PET	Planar	75, 150	2.0		300 s	[68]
COC	Roller	70–110	0.55		0.5 m/min	[69]
COC	Planar	160	0.62		5 min for thermal equilibrium 30 s for press	[70]
COC	Planar	150	5		300 s	[71]
PEEK	Planar	180–280		20 k	10 min	[72]
PEEK	Planar	365		25 k	1 min	[73]
PC	Planar	160	5		300 s	[74]
PC	Planar	180	10		10 min	[75]
Epoxy	Planar	95	1.2		10 min	[76]
PI	Planar	200	3		2 min	[77]
CNF	RTR	155	8.3		0.3 m/min (imprint time 1 s)	[62]
FEP	Planar	270	0.18		5 min	[78]
ETFE	Planar	250	1.38 3.10		10 s 1 min	[79]
PEI	Planar	285	1.0		3 min	[80]
CA	RTR	115	13.6		0.2 m/min	[81]
PSU	RTR	155		300 N/mm	2.1 m/min	[82]
PES	RTR	166		300 N/mm	2.1 m/min	[82]
PVC	Planar	<120	<1		N/A	[83]

PMMA, Poly(methyl) methacrylate; PS, Polystyrene; PEN, Polyethylene naphthalate; PE, Polyethylene; PP, Polypropylene; PET, Polyethylene terephthalate; COC, Cyclic olefin copolymer; PEEK, Polyether ether ketone; PC, Polycarbonate; PI, polyimide; CNF, Cellulose nano fiber; FEP, Fluorinated Ethylene Propylene; ETFE, Ethylene tetrafluoroethylene; PEI, Polyetherimide; CA, Cellulose Acetate; PSU, Polysulfone; PES, Polyethersulphone; PVC, Polyvinyl chloride.

## Data Availability

Not applicable.
